# Estimating the combined costs of clinical and subclinical ketosis in dairy cows

**DOI:** 10.1371/journal.pone.0230448

**Published:** 2020-04-07

**Authors:** Wilma Steeneveld, Paul Amuta, Felix J. S. van Soest, Ruurd Jorritsma, Henk Hogeveen

**Affiliations:** 1 Department of Farm Animal Health, Faculty of Veterinary Medicine, Utrecht University, Utrecht, the Netherlands; 2 Chair group Business Economics, Wageningen University, Wageningen, the Netherlands; Michigan State University, UNITED STATES

## Abstract

Clinical ketosis (CK) and subclinical ketosis (SCK) are associated with lower milk production, lower reproductive performance, an increased culling of cows and an increased probability of other disorders. Quantifying the costs related to ketosis will enable veterinarians and farmers to make more informed decisions regarding the prevention and treatment of the disease. The overall aim of this study was to estimate the combined costs of CK and SCK using assumptions and input variables from a typical Dutch context. A herd level dynamic stochastic simulation model was developed, simulating 385 herds with 130 cows each. In the default scenario there was a CK probability of almost 1% and a SCK probability of 11%. The herds under the no risk scenario had no CK and SCK, while the herds under the high-risk scenario had a doubled probability of CK and SCK compared to the default scenario. The results from the simulation model were used to estimate the annual cash flows of the herds, including the costs related to milk production losses, treatment, displaced abomasum, mastitis, calf management, culling and feed, as well as the returns from sales of milk and calves. The difference between the annual net cash flows of farms in the no risk scenario and the default scenario provides the estimate of the herd level costs of ketosis. Average herd level costs of ketosis (CK and SCK combined) were €3,613 per year for a default farm and €7,371 per year for a high-risk farm. The costs for a single CK case were on average €709 (with 5 and 95 percentiles of €64 and €1,196, respectively), while the costs for a single SCK case were on average €150 (with 5 and 95 percentiles of €18 and €422, respectively) for the default farms. The differences in costs between cases occurred due to differences between cases (e.g., cow culled vs cow not culled, getting another disease vs not getting another disease).

## Introduction

High producing dairy cows experience a negative energy balance following parturition. This negative energy balance results from a fast increase in energy requirements for milk production while feed intake capacity in early lactation is limited. Subclinical ketosis (SCK), generally defined as a blood betahydroxybutyrate (BHBA) concentration of 1,4 μmol/L or higher during early lactation without clinical signs such as a disappointing milk production with an elevated fat and a lowered protein content [1], can occur if blood ketone bodies increase dramatically. A few publications have reported thresholds between 1,2 up to 1,4 μmol/L [2]. In some cases, cows with elevated concentrations of ketone bodies show clinical signs and are thus considered to have clinical ketosis (CK). Berge and Vertenten [3] reported an average herd prevalence of CK varying between 0.7 and 3.5%. The herd prevalence of SCK depends on the BHBA cut-off used and on the method for herd selection. For example, a random herd selection in the Netherlands by Van der Drift et al. [4] resulted in a prevalence of SCK (defined as plasma BHBA ≥1,200 μmol/L) of 11.2%, while a convenience sample by Berge and Vertenten [3] resulted in an SCK prevalence of 48% (defined as milk BHBA ≥100 μmol/L).

Elevated concentrations of ketone bodies in blood are associated with a lower milk production and reproduction performance, and an increase in the culling of cows (e.g., [5]). While the level of the effects of a case of CK are in general more severe than for a case of SCK, they are both also associated with an increased probability of other disorders such as displaced abomasum (DA), cystic ovary and mastitis (e.g., [3, 6]), and increased veterinary and treatment costs (e.g., [6–7]).

Quantifying the losses due to ketosis will enable veterinarians and farmers to make more informed decisions regarding the prevention and treatment of the disease. Some studies compared the economic consequences of various treatment strategies [7–9], whilst many more studies estimated the economic consequences of SCK and CK [9–14]. Raboisson et al. [11] and Mostert et al. [12] estimated average costs for a cow with SCK at €257 and €130 respectively. These differences are probably due to the use of other model types and also different assumptions and input values. For instance, Raboisson et al. [11] developed a static model, while Mostert et al. [12] developed a dynamic model and thus simulated milk production, reproduction and culling over time. Also, the studies modeled the effect of SCK on culling differently. In Mostert et al. [12] the culling of cows was only possible at day 30 of lactation, which probably resulted in an underestimation of the culling costs. Van Soest et al. [13] estimated the costs of ketosis at €21 per cow per year. Only Liang et al [14] focused on the costs of CK only. Other studies [11–13] didn’t differentiate between the costs of CK and SCK. It seems justified to differentiate between SCK and CK as it is likely clinical cases are more often treated than subclinical cases.

The overall aim of this study was to estimate the combined costs of CK and SCK, using assumptions and input variables from a typical Dutch context. A further aim was to evaluate the economic consequences of five different treatment strategies for CK and SCK. For these purposes, a dynamic stochastic simulation model at herd level was developed.

## Materials and methods

The biological cow simulation model was developed using Microsoft Excel with @Risk add-in software (2002; Palisade Corp., Newfield, NY). The model used in this study is an adaptation and extension of the cow simulation model described by Inchaisri et al. [15] and Rutten et al. [16] and simulates an individual cow place which is filled with a dairy cow for which all relevant events occur in weekly time steps. The cow simulation model specifications are described below, with a specific emphasis on the new components. The new model components include the occurrence of CK and SCK, DA and clinical mastitis, as well as the effect of CK and SCK on reproduction performance, culling and milk production. A formal description of the new components in the model is provided in the supporting information.

### Simulation model

The cow simulation model simulates a single cow place. In this study, each iteration of a cow place started with a cow at the point of calving with a randomly assigned parity (based on a parity distribution used by Inchaisri et al. [15]) and a relative milk production factor. The model followed the cow in weekly time steps after calving until she was culled. Once a cow was culled, she was replaced by a replacement heifer with its own relative milk production factor. The cow simulation model followed a cow place for a total of 16 consecutive lactations to obtain model stability. Input values related to the occurrence of CK and SCK are presented in Table 1.

**Table 1:** Input values for cow factors and their relation with clinical ketosis (CK) and subclinical ketosis (SCK) for the default scenario.

Parameter	Value	Source
Lactational probability of cows with SCK	0.11	[4]
Lactational probability of cows with CK	0.007	[3]
Transition parameter from SCK to CK	0.04	Authors’ expertise
Annual probability of both CK and SCK occurring ≤ 4 weeks in milk	0.75	[5, 17]
Annual probability of SCK cases being treated	0.1	Authors’ expertise
Annual probability of CK cases being treated	1	Authors’ expertise
Treatment effect, proportion reduction in milk production losses	0.5	[18]
Percentage milk production loss, SCK	6% - 8%	[1, 2, 5]
Percentage milk production loss, CK	15% - 17%	[19]
Probability of culling a cow with CK in the 4 weeks following diagnosis	0.3	Authors’ expertise
Probability of culling a cow for fertility	0.2	[16]
Annual probability of cows with displaced abomasum	0.011	[6]
Annual probability of cows with clinical mastitis	0.3	[6, 20]
Relative risk displaced abomasum	5	[1, 2, 3]
Relative risk clinical mastitis	1.3	[2, 3]

#### Milk yield

At the start of each lactation, the potential 305-day milk production for each cow was determined based on the average herd milk production of a Dutch dairy farm (8,742 kg/305 d [21]), a relative milk production factor and an assigned parity. Subsequently, the associated potential lactation curve of each cow was determined using Wood’s function [22]. After successful conception, a reduction in potential daily milk production was calculated until drying off at 60 days before the associated calving date. A further reduction in milk production was calculated for the cows that developed SCK or CK.

Based on the simulated lactation curve, feed requirements as energy requirements, expressed in feed units for lactation (VEM; 1 VEM = 1.65 kcal of NEL) as defined by Van Es [23] were calculated (as described in [16]). Higher feed requirements were simulated for first- and second-parity cows, and for different pregnancy stages [16].

#### CK and SCK

The model simulated three scenarios. In the default (base risk) scenario there was a CK a probability of 0.007 and an SCK probability of 0.11 per cow per lactation. The herds under the no risk scenario had no CK and SCK, while the herds under the high-risk scenario had a doubled probability of developing CK and SCK compared to the default scenario. For the default and high-risk scenarios it was assumed that SCK and CK occurred not more than once per lactation. The probability of developing SCK and CK was adjusted according to the relative milk production factor and lactation stage in such a way that cows with a relative high milk yield were more likely to develop SCK or CK. The increased probability of SCK cows becoming CK cows (details in S1 File) was also included. In total, 75% of all SCK and CK cases occurred in the first four weeks of lactation and 50% of all CK cases were linked to SCK and occurred within one week of developing SCK. The remaining CK cases occurred without a preceding SCK case.

Milk production loss associated with CK and SCK was calculated based on daily milk yield and corrected for conception status. The milk production loss due to SCK and CK, expressed as a percentage of the daily milk yield, was drawn from an uniform distribution with a minimum and maximum of 6% and 8% for SCK and 15% and 17% for CK. The production loss is highest in the week of occurrence of either CK or SCK, afterwards milk production losses decrease with an adjustment factor (0.7) and is pruned to 0 when milk production losses in the previous week drop below 1.5%.

Simulation of SCK and CK treatments resulted in a 50% recovery of the milk production losses due to SCK or CK. A cow with SCK had a 0.1 probability of being treated, with treatment always taking place in the same week that SCK occurred. A cow with CK was always treated immediately. As some cases may need veterinary intervention, the simulation included a veterinary visit and consecutive treatment for SCK and CK with a probability of 0.5 and 1.0 respectively.

#### Increased probability of other disorders

In each week, the occurrence of clinical mastitis depended on the ketosis status of the cow and was adjusted for the cow’s week in milk in such a way that 70% of the mastitis cases occurred in the first four weeks of lactation and 30% in a later stage. A relative risk of 1.3 was included in the model for the effect of SCK on the occurrence of mastitis. The probability of DA occurrence depended on the ketosis status of the cow in that week and was adjusted for the cow’s week in milk in such a way that 75% of DA cases occurred in the first four weeks of lactation and 25% in a later stage. The probability of DA occurrence depended on a relative risk of 5.0 of cows having SCK.

#### Reproductive cycle

Modelling of the reproductive cycle is described by Inchaisri et al. [15], and for the current study the effects of CK and SCK are added. In short, the gestation length (in weeks after conception) and dry off date (gestation length minus eight weeks) were determined at the start of each lactation. Furthermore, the simulation took the effects of CK and SCK on fertility into account. CK and SCK postponed ovarian cyclicity, reduced conception rates and caused embryonic death. Detection of estrus in a certain week depended on the occurrence of ovulation, the relative milk production factor, peak milk yield and milk yield such that a cow with a relatively low milk yield was more likely to be detected and vice versa. A cow was inseminated at the first detected estrus after a voluntary waiting period of 12 weeks, which was repeated for the following detected estrus if the preceding conception was modeled as unsuccessful. The basis conception rate was 50%, and conception was adjusted for parity, relative milk production factor, occurrence of postpartum disorders (e.g., metritis) and occurrence of CK and SCK. It was assumed that CK and SCK decreased conception rates by 20% when ketosis was present in the previous week. For cows without CK or SCK the conception rate was increased by 3% [2].

#### Culling of cows

Cows were culled either as a result of poor fertility, CK or for other reasons. Modelling of culling did not differ between the scenario’s (default, no risk, high risk). If a cow was not pregnant at either 35 weeks after parturition or after six inseminations, she was culled for fertility reasons. Cows were also culled if their daily milk production was below 15 kg milk/d. On average, 20% of all culled cows were culled for fertility reasons at a replacement rate of 30%. Cows that developed CK had a probability of 0.31 of being culled in the following four weeks. The probability of culling due to SCK was modeled indirectly as cows with SCK had a reduced conception rate and consequently a higher probability of being culled due to poor fertility. The probability of culling due to other reasons was determined in such a way that the final culling rate was 30%. To make sure that the final culling rate was 30% an adjustment factor was used (see S**1**File).

### Model outputs

In the model, the herds consisted of 130 cows each. Every simulation included 385 herds, each with 130 cow places. A simulation is defined as running the model for 385 herds with 130 cows each, so it consists of 50,050 iterations, each iteration resembling 1 cow. Each described scenario required one simulation. For every simulation, the model ran 50,050 iterations, with each iteration representing one cow place for 16 consecutive parities. 50,000 iterations were needed to achieve stability of the simulation results (if the model was run again the results will be the same) and an additional 50 iterations were added to have a uniform number of cows in each of the 385 herds. The output values were always stable before the 5^th^ year, so the output values of the 6^th^ year were used as the model output. To minimize the total runtime of the simulation as well as to get sufficient information on the variation in economic effects of ketosis at the herd level, 385 herds of 130 cows each were simulated. The choices are made to represent the Dutch situation, regarding herd size, herd management and ketosis situation. A herd size of 130 cows was chosen to represent a future Dutch dairy herd with two milking robots (see Rutten et al. [16], whose simulation model is adapted.

The model outputs for each iteration (cow place) included annual milk production, the annual number of CK and SCK cases, annual milk production losses due to CK and SCK, the annual number of calves born, the annual number of inseminations, the annual number of DA cases, the annual number of clinical mastitis cases, the annual number of culled cows and the annual feed intake measured in VEM/year [16].

### Economic output

In order to investigate the costs of ketosis, the annual model outputs were used to calculate cash flows for milk produced, calves sold, treatment costs, DA costs, clinical mastitis costs, calf management costs, culling costs and feed intake costs for all three scenarios (no risk, default, and high risk). Input values were based on the 2018 Dutch market context (Table 2).

**Table 2:** Default input values of costs and prices used in the economic analysis for costs of ketosis, and their sources of origin.

Parameter	Value	Unit	Source
Milk production			
Milk price	0.35	€/kg	[24]
Feed costs	0.16188	€/ kVEM^a^	[25]
Treatment costs			
Medication costs	16.5	€/case	Expert opinion
Labor costs	24	€/hour	[24]
Time spent per case	1.5	hours	Expert opinion
Diagnostics	1	€/case	Expert opinion
Veterinary service costs	70	€/case	Expert opinion
Costs of other diseases/reproduction			
Treatment costs of displaced abomasum	125	€/case	Expert opinion
Treatment costs of clinical mastitis	30	€/case	Adapted from [26]
Insemination costs	30	€/insemination	Dutch market price
Calf management costs	180	€/calving	Authors’ expertise
Calf price	75	€/calf	Dutch market price
Slaughter prices			[16, 27]
Parity 1	668	€/cow	
Parity 2	753	€/cow	
Parity 3	779	€/cow	
Opportunity cost replacement heifer^b^	965	€/heifer	[27]

^a^VEM is the feed requirements estimated as energy requirements in feed units for lactation, as defined by Van Es [23].

^b^Represent the market value of a retained heifer that could have been sold at weaning.

#### Costs of milk production losses due to SCK and CK

The costs of milk production losses due to SCK and CK were calculated based on the level of milk production losses, the milk price and the reduced feed costs associated with reduced milk production as a result of CK and SCK, respectively.

#### Costs of SCK and CK treatments

Treatment costs of ketosis included the costs for diagnostics and drugs, veterinary visits and the farmer’s labor costs. It was assumed that all cases of CK needed a veterinary visit, a diagnostic work-up, the application of drugs by a veterinarian and labor input from the farmer (Table 2). Total treatment costs for a single CK case will be €123.5 (medication €16.5, diagnostics €1, veterinary service costs €70, and labor 1.5 hours for €24 per hour). It was assumed that only 20% of SCK cases were detected and that 50% of those received veterinary attention. Therefore, only 10% of SCK cases resulted in diagnostics, drugs, veterinary visits and farmer’s labor costs.

#### Fertility costs

The costs associated with impaired fertility as a result of ketosis included the costs of insemination, calf management and calf sales. Insemination costs were calculated as the annual number of inseminations in the herd multiplied by the cost per insemination. The price of insemination included the costs of semen, the insemination service and the farmer’s labor [16]. The costs for calf management and calf sales were calculated as the number of calves born in the herd per year multiplied by the management costs per calving and the price of a calf.

#### Costs of displaced abomasum and clinical mastitis

DA and clinical mastitis were associated with costs for milk production losses, increased culling probability and treatment costs. Costs for milk production losses and culling were already calculated for the costs of SCK and CK. To avoid double counting, only the treatment costs were calculated for DA and clinical mastitis.

#### Costs of culling

The net culling costs included the slaughter value of the culled cow (revenue) and the opportunity costs of the replacement heifer (cost). These values were calculated using the same approach as Rutten et al. [16]. Slaughter prices and the sales price of heifers were updated, and based on prices in the period 2010 to 2017 [27]. The net costs of culling were €297, €212, and €186 for a cow in first, second and third, or higher parity, respectively (Table 2). The costs of culling were independent on lactation stage.

#### Costs of ketosis

For all three scenarios (no risk, default, high risk) the annual net cash flow on a farm was calculated as the sum of cash flows from milk production and calves sold, and costs for treatment, DA, clinical mastitis, calf management, culling and feed. The annual net cash flows for all 385 farms in the simulation were averaged. The difference between the average annual net cash flows for the no risk scenario and the default scenario was calculated to represent the overall costs of ketosis (CK and SCK) for a 130-cow dairy farm in the default scenario. In addition, the difference between the average annual net cash flows for the no risk scenario and the high-risk scenario was calculated to represent the overall costs of ketosis (CK and SCK) for a 130-cow dairy farm in the high-risk scenario.

The costs for a single CK and SCK case were calculated in two steps. First, the costs of milk production losses and treatment for CK and SCK were divided by the number of cases of CK and SCK, respectively. Subsequently, all other costs were split equally over all cases (CK and SCK alike). Both values were added up for CK and SCK to calculate the costs for a single case of CK and SCK, respectively. The costs in the default scenario were used for this calculation.

### Treatment strategies

The economic consequences of different treatment strategies were determined by comparing five different treatment strategies. The default strategy (i) includes the situation described in the default scenario model where all CK cases and 10% of the SCK cases are treated. The four alternative treatment strategies are: (ii) a strategy in which all cases of ketosis (CK and SCK) are treated, (iii) a strategy in which all CK cases and half of the SCK cases are treated, (iv) a strategy in which all CK cases are treated and none of the SCK cases are treated and (v) a strategy in which no case of ketosis (CK or SCK) is treated. These different scenarios are selected to study in particular the impact of treatments strategies, while they may also reflect the approach of farmers that are either reluctant or focused to diagnose and treat each diseased cow.

### Validation and sensitivity analysis

Because data were not available for external validation of the model, an internal validation was performed. A large number of inputs were compared with the output to check the consistency and the credibility of the model output.

A sensitivity analysis was conducted to assess the level of impact of some input parameters on the overall costs of ketosis in the default scenario. Values for input variables in the sensitivity analysis were based on information in the literature and assumptions made by the authors. Sensitivity analyses were performed for the annual probability of CK and SCK, percentage milk production losses for CK and SCK and average herd milk production. In Table 5 the values used in the sensitivity analyses are presented.

## Results

### Biological output

The average simulation results of the 385 herds of 130 cows for the no risk, default and high-risk scenarios are presented in Table 3. In the default scenario, the average annual milk production loss due to two CK cases was 482 kg, while the average annual milk production loss due to 16 SCK cases was 2,768 kg. The average losses per SCK and CK case are thus approximately 173 and 241 kg, respectively. In the high-risk scenario, the average annual milk production loss due to six CK cases was 1,199 kg, while the average annual milk production loss due to 37 SCK cases was 6,126 kg. In addition, there was an increase in the annual average number of DA cases from one (no risk scenario) to two (default scenario and high-risk scenario), an increase in the annual average number of clinical mastitis cases from 42 (no risk and default scenario) to 45 (high-risk scenario), an increase in the annual average number of inseminations from 267 (no risk scenario) to 275 (default scenario) to 280 (high-risk scenario) and an increase in the annual average number of culled cows from 38 (no risk scenario) to 39 (default scenario) and 40 (high-risk scenario).

**Table 3:** Average (with 5^th^ and 95^th^ percentiles) annual technical simulation results for farms with 130 dairy cows in the no risk, default and high-risk scenarios. Values are rounded to the nearest whole number (except for displaced abomasum). CK = clinical ketosis, SCK = subclinical ketosis.

	No risk	Default	High risk
Milk production (x 1,000 kg)	1,156 (1,129; 1,191)	1,148 (1,139; 1,167)	1,144 (1,129; 1,157)
Milk production (kg/cow)	8,891 (6,808; 10,929)	8,814 (6,806; 10,887)	8,786 (6,823; 10,991)
Feed (x 1,000 kVEM^a^)	908 (891; 935)	903 (895; 920)	901 (897; 915)
CK cases (no.)	0	2 (0; 5)	6 (4; 14)
Total SCK cases (no.)	0	16 (16; 28)	37 (32; 48)
CK milk losses (kg)	0	482 (403; 811)	1,199 (756; 1,547)
SCK milk losses (kg)	0	2,768 (2,215; 3,845)	6,126 (4,395; 7,142)
Displaced abomasum (no.)	0.9 (0; 2)	1.5 (0; 3)	1.8 (0; 3)
Clinical mastitis (no.)	42 (31; 49)	42 (37; 50)	45 (38; 49)
Inseminations (no.)	267 (202; 288)	275 (215; 311)	280 (228; 345)
Calves (no.)	145 (135; 151)	142 (137; 153)	144 (130; 149)
Calving interval (d)	400 (360; 478)	403 (361; 480)	408 (364; 482)
Culling (no.)	38 (34; 51)	39 (32; 54)	40 (35; 54)

^a^VEM is the feed requirements estimated as energy requirements in feed units for lactation, as defined by van Es (1978).

### Economic output

**Fig 1:**
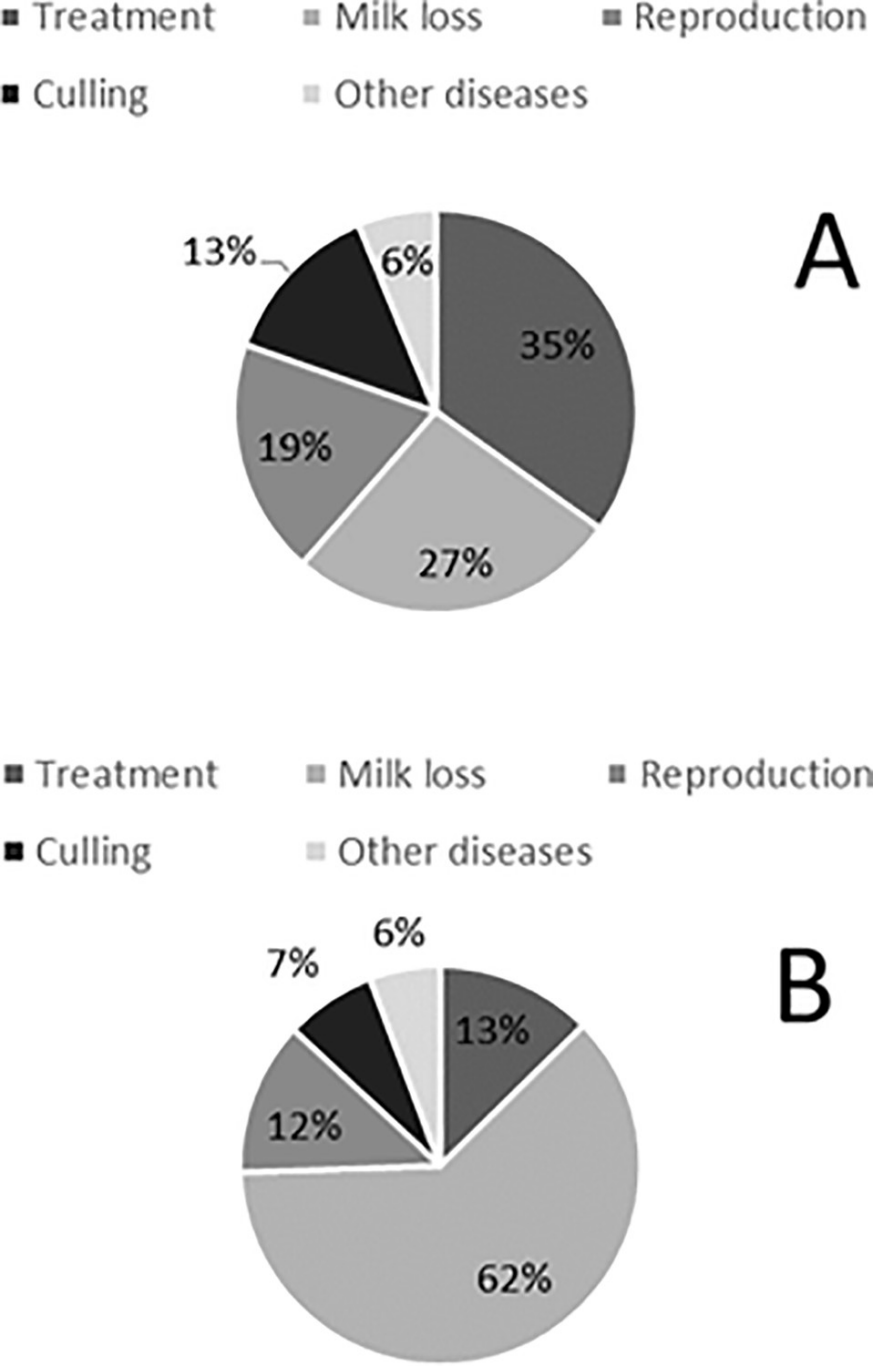
Percentage contribution to the total costs of a single clinical ketose (A) and subclinical ketose (B) case.

The annual economic consequences of the simulation results are presented in Table 4. The overall costs for CK and SCK for a 130-cow dairy farm were €3,613 per year in the default scenario and €7,371 per year in the high-risk scenario. Milk revenues were higher in comparison to the default and high-risk scenarios when there was no ketosis. Furthermore, culling contributed to the overall costs of ketosis, decreasing the overall costs by €170 in the default scenario and by €231 in the high-risk scenario. Costs for milk production losses due to CK in the default and high-risk scenarios were €169 and €420, respectively. Costs for milk production losses due to SCK in the default and high-risk scenarios were €1,175 and €2,144, respectively. Ketosis also caused higher insemination costs and costs for other diseases (DA and clinical mastitis) in comparison to the no risk scenario. In addition, the annual treatment costs were €221 (default scenario) and €711 (high-risk scenario) for CK, and €198 (default scenario) and €457 (high-risk scenario) for SCK. The costs for a single CK case were on average €709 (with 5 and 95 percentiles of €64 and €1,196), while the costs for a single SCK case were on average €150 (with 5 and 95 percentiles of €18 and €422, respectively) in the default scenario. The contribution due to milk loss, treatment, reproduction, culling and other diseases to the costs of a single case of CK and SCK are presented in Fig 1A and 1B. For CK, treatment and milk loss had the highest contribution to the costs of a single case. For SCK, milk loss had the highest contribution to the costs of a single case.

**Table 4:** Average (with 5^th^ and 95^th^ percentiles) annual net cash flows (€) based on the technical simulation results for a farm with 130 dairy cows with no risk of ketosis, a default risk of ketosis and a high risk of ketosis. The overall costs of ketosis were calculated for the default and the high-risk scenarios. Values are rounded to the nearest whole number. CK = clinical ketosis, SCK = subclinical ketosis.

	No risk	Default	High risk	Difference in cash flow (default scenario vs no risk scenario)	Difference in cash flow (high-risk scenario vs no risk scenario)
Milk production revenues (€)	404,539 (395,467; 416,643)	401,875 (398,503; 408,394)	400,420 (395,097; 405,109)	2,664	4,119
Feed costs (€)	146,983 (144,192; 151,087)	146,179 (146,052; 148,987)	145,798 (145,325; 148,159)	804	1,185
CK milk loss (€)	-	169 (141; 284)	420 (265; 540)	-169	-420
SCK milk loss (€)	-	969 (785; 1,241)	2,144 (1,699; 2,718)	-969	-2,144
CK treatment (€)	-	221 (208; 894)	711 (480; 1,666)	-221	-711
SCK treatment (€)	-	198 (174; 312)	457 (373; 564)	-198	-457
Displaced abomasum treatment (€)	112 (0; 288)	188 (0; 375)	225 (0; 413)	-76	-113
Clinical mastitis treatment (€)	1,269 (928; 1,471)	1,272 (1,109; 1,498)	1,335 (1,138; 1,469)	-3	-66
Inseminations (€)	8,010 (6,061; 8,638)	8,250 (6,449; 9,327)	8,400 (6,841; 10,347)	-240	-390
Calf management costs (€)	26,089 (20,170; 22,876)	25,558 (20,778; 23180)	25,916 (19,821; 22,633)	531	173
Culling costs (€)	8,745 (6,014; 8,985)	8,915 (6,245; 9,023)	8,976 (6,378; 9,562)	-170	-231
Calf sales (€)	10,880 (13,270; 15,050)	10,642 (13,670; 15,250)	10,802 (13,040; 14,890)	238	78
Total (€)	224,211 (208,760; 245,549)	220,598 (202,986; 240,452)	216,840 (197,953; 236,982)	3,613^a^	7,371^b^

^a^Overall total costs calculated as the sum of the differences in cash flows (2,664+169+969+221+198+76+3+240+170+238-804-531)

^b^Overall total costs calculated as the sum of the differences in cash flows (4,119+420+2,144+711+457+113+66+390+231+78–1,185–173)

The overall costs of ketosis when treating all SCK and CK cases were €4,348 per year. Treating all CK cases and half of the SCK cases resulted in overall ketosis costs of €1,051 per year, while treating all CK cases and no SCK cases resulted in overall ketosis costs of €1,471 per year. Treating no ketosis cases resulted in overall ketosis costs of €2,098 per year.

The sensitivity analysis shows that the overall total costs of ketosis are most sensitive to the probability of getting CK and SCK. Varying the milk production loss had only a small effect on the overall costs (Table 5).

**Table 5:** Sensitivity of the overall costs of ketosis (€/year) for a higher and lower value of different input values. Values for overall costs are rounded to the nearest whole number. CK = clinical ketosis, SCK = subclinical ketosis.

Input	Value	Default	Overall costs (€/year)
Annual probability of CK	0.0035	0.007	3,271
	0.014	0.007	6,124
Annual probability of SCK	0.055	0.11	2,942
	0.22	0.11	6,985
% production losses due to CK	11–14	15–17	3,816
	18–20	15–17	3,864
% production losses due to SCK	3–5	6–8	3,815
	9–11	6–8	3,861
Average herd milk production (kg/year)	8,000	8,742	3,643
	9,500	8,742	3,974

## Discussion

The results of the current study differ from results obtained from other studies. For instance, Geishauser et al. [8] estimated the costs of SCK at US$78 (approximately €67). The difference seem mostly due to different assumptions and input values. The Geishauser et al. [8] study, may, for example, have underestimated the losses as the researchers excluded the increased probability of culling due to ketosis, did not take into account treatment and calculated milk losses for only two weeks (2 kg per day). In our study, a carry-over effect that ensured that milk losses were calculated for 2–6 weeks following a ketosis event was incorporated, resulting in approximately 173 kg loss per SCK case (see Table 3, 2768 kg for 16 SCK cases). Liang et al. [14] developed a stochastic Monte Carlo model and is one of the few studies that estimated the costs of a CK case ($77 (approximately €67) for a primiparous cow and $181 (approximately €158) for a multiparous cow). Comparing with Liang et al. [14] seems difficult because of very different assumptions on milk production loss (much lower effects in [14]), reproduction (different for heifers and multiparous cows in [14]) and other diseases (not taken into account in [14]). McArt et al. [10] developed a deterministic model and estimated the costs of SCK at US$289 per case (approximately €250). Mostert et al. [12] estimated the costs of SCK at €130 per case with a dynamic stochastic Monte Carlo simulation model. The main difference with the current study is that Mostert et al. [12] assumed that all culling occurred on day 30 of lactation. The culling of cows in a later lactation stage (e.g. because of reproduction problems due to SCK) was not included in [12], possibly resulting in an underestimation of the culling costs. It is important to include all ketosis costs in a cost estimation, as farmers adjust their management based on these estimates. Recently, Van Soest et al. [13] developed a calculation tool to estimate the costs of production diseases, including ketosis. Based on data from European organic farms, this tool estimates that the average costs of ketosis were €21 per cow per year, but this figure varied highly between farms. Therefore, it is important to make farm-specific estimations on the costs of ketosis in order to aid decision-making relating to on-farm support for ketosis [13].

The current study developed a stochastic simulation model for the estimation of the costs of ketosis, thus making the variation in results available as well (Tables 3 and 4). The costs for a single SCK case varied between €15 and €418 (5 and 95 percentiles) for a herd with a default risk of ketosis and may illustrate the size of variation in economic loss due to SCK encountered in daily practice. Such a variation has also been shown previously [e.g., 12, 13]. Van Soest et al. [13] observed differences between European countries, but observed even larger differences in ketose prevalences between herds within countries. Moreover, in literature prevalences of ketosis might have been underreported, and probably prevalences and thus costs are much higher. Therefore, a higher probability of ketosis was included in the sensitivity analysis. Herds with a high probability of getting ketosis have much higher ketosis costs (Table 5). A lower or higher average herd milk production only had a minor effect on the costs of ketosis. The effect of herd size on the costs of ketosis was not investigated as the herd size, as such, is important for the variation in yearly costs due to ketosis, and not for the average. With smaller herd sizes, the average outcomes will not change (average costs of ketosis per herd per year as well as average costs of a CK or SCK case). However, because of the numbers involved, the variation between farms will change.

The estimated milk production losses in the current study may have been under- or overestimated because it was difficult to understand the pattern of milk losses due to ketosis (e.g., [1–2, 19]) and the effect of treatment on milk production. Therefore, milk production losses were included in the sensitivity analysis and the results show that changing milk production losses have only a minor effect on the overall costs of ketosis. For instance, an increase in milk production losses due to SCK by 3% increased the overall costs of ketosis by only €46 per year (Table 5). It is very difficult to obtain a good estimate of the real effect of SCK on milk production losses as this would require a follow-up of the affected cows during an entire lactation period. Such a study would be complicated by the fact that milk production losses also occur due to other conditions such as pregnancy, mastitis, lameness and DA, making it tempting to assign a certain milk production loss to each of these conditions. Therefore, in the current study a combined estimation of the milk production losses was calculated and attributed to SCK and CK. Costs for clinical mastitis and DA consisted therefore only of treatment costs in order to avoid double counting. McArt et al. [10] did include milk production losses due to DA in the cost estimation of SCK, and found total costs of US$289 per case. This value is higher than the €150 found in the current study, and this is probably a result, in our opinion, of an overestimation of milk production losses.

In the no risk scenario, annual milk production (Table 3) was higher compared to the default and high-risk scenarios. This was the result of cows having a shorter calving interval in the no risk scenario (Table 3), which in turn resulted in higher milk production per year. Previous studies reported that a shorter calving interval resulted in higher annual milk production (e.g., [15, 28]). These studies included fixed values for this reproduction effect [9] or did not include it at all [10]. The current dynamic stochastic simulation model included the complex interactions of disease and production processes under various conditions, taking into account the variation associated with biological systems, as well as the time sequence of events. This made it possible to demonstrate that impaired reproduction resulted in more inseminations, fewer calves, longer calving intervals and earlier removal from the herd, over a period of weeks, months and years.

In the current model it was decided that the overall replacement rate was kept at 30%, independent of the ketosis situation. As a consequence, cows with ketosis were sometimes culled whereas cows without ketosis (which otherwise would have been culled) were kept longer. The costs of culling therefore represent the costs for a less optimal culling policy. Although this is not optimal from an economic point of view, it largely represents the approach many farmers use in practice and was also used by Rutten et al. [16]. This way of modeling culling may therefore provide more realistic results than the assumption of optimal culling decisions used in other models (e.g., [29]). As a consequence, costs of culling in the current study are lower than in studies that assumed optimal culling decisions, e.g., [30].

A few other studies investigated the economic consequences of different treatment strategies. They compared treatment strategies in combination with the testing of cows suspected of having SCK [7–9]. The current study performed a comparison of the different treatments after SCK or CK was diagnosed in order to reflect the current situation in Dutch dairy herds. Results showed that the treatment strategy in which all CK cases and half of the SCK cases were treated was economically the most attractive. Treating all CK and SCK cases was economically the least attractive strategy. This outcome depends, of course, on different input values and may vary between countries.

## Conclusions

Average herd level ketosis costs (CK and SCK combined) were €3,613 per year for a default farm and €7,371 per year for a farm in the high-risk scenario. The costs for a single CK case were on average €709 (with 5 and 95 percentiles of €64 and €1,196, respectively), while the costs for a single SCK case were on average €150 (with 5 and 95 percentiles of €18 and €422, respectively) for the default farm. Results of the sensitivity analysis show that the probability of CK and SCK have the largest effect on the herd level ketosis costs and that using other percentages for production losses for CK and SCK had a relatively small effect on the herd level ketosis costs. Because of the high impact of the probability of ketosis at the herd level, from an economic point of view the prevention of ketosis on high-risk farms is an attractive option.

## Supporting information

S1 File (DOCX)
