# Supplementary material for: Estimating the combined costs of clinical and subclinical ketosis in dairy cows
Source: PLoS One. 2020 Apr 7;15(4):e0230448. doi: 10.1371/journal.pone.0230448 (PMC7138322; doi:10.1371/journal.pone.0230448)
Supplement: S1 File — (DOCX) [file pone.0230448.s001.docx]

**Supporting information**

This appendix provides a description of the model, and includes more mathematical detail than the Material and Methods section.

The cow simulation model was developed in Microsoft Excel software. For the stochastic properties of the model, the add-in software @Risk 6 for Excel (Palisade Corporation, 2010) was used. New components of the cow simulation model are described in detail in this appendix, and include the occurrence of CK and SCK, displaced abomasum, and clinical mastitis. In addition, the effect of clinical ketosis (CK) and subclinical ketosis (SCK) on fertility, culling, and milk production are described. An overview of model inputs is given in Table S1, which is an extended version of Table 1. The basic specifications of the model were already described by Inchaisri et al. (2010) and Rutten et al. (2014).

**Occurrence of ketosis**

The risk of subclinical ketosis (R_SCKijp_) of cow *i*, in parity *p* and week *j* is determined as follows:

$$R_{SCKijp}= \left\{ \begin{aligned} if {MP}_{ijp}<20 then 0 \\ if j>1 and max{SK}_{ijp}:{SK}_{ij-1p}>0 then 0 \\ if j\leq4 then R_{HS}\times{AdjMP}_{ip}\times\frac{R_{WIM\leq4}}{4} \\ else R_{HS}\times{AdjMP}_{ip}\times\frac{R_{WIM>4}}{28} \\ i=1,\ldots n \\ j=1,\ldots, week at drying off \\ p=1,\ldots, n \end{aligned} \right.$$

where *MP_ijp_* is the milk production of cow *i*, in parity *p* and week *j* (explained in Inchaisri et al. 2010), and SK*ij-1p* the status of ketosis in the previous WIM.

R_HS_ is the transition parameter from healthy to subclinical ketosis, and AdjMP_ip_ is the adjustment factor which assumes that cows with a higher milk yield are at a higher risk of getting ketosis. R_WIM≤4_ and R_WIM>4_ are the annual proportions of ketosis that occur before and after four WIM, respectively. Division by 28 is because on average milk production drops below 20kg/day at *j*=28.

The risk of clinical ketosis (R_CKijp_) of cow *i*, in parity *p* and week *j* is determined as follows:

$$R_{CKijp}= \left\{ \begin{aligned} if {MP}_{ijp}<20 then 0 \\ if j>1 and max{SK}_{ijp}:{SK}_{ij-1p}=2 then 0 \\ if j=2 and {SK}_{ij-1p}=2 then R_{SC}\times{AdjMP}_{ip} \\ if j>2 and max{SK}_{ij-2p}:{SK}_{ij-1p}=1 then R_{SC}\times{AdjMP}_{ip} \\ if j>1 and max{SK}_{ijp}:{SK}_{ij-1p}>0 then 0 \\ if j\leq4 then R_{HC}\times{AdjMP}_{ip}\times\frac{R_{WIM\leq4}}{4} \\ else R_{HC}\times{AdjMP}_{ip}\times\frac{R_{WIM>4}}{24} \\ i=1,\ldots n \\ j=1,\ldots, week at drying off \\ p=1,\ldots, n \end{aligned} \right.$$

Where R_SC_ is the transition parameter from SCK to CK, and R_HC_ is the transition parameter from healthy to CK.

The ketosis status (SK_ij_) of a cow *i*, in parity *p* and week *j*, is determined for every week in milk (WIM) as a discrete event:

$${SK}_{ijp}=Discrete \left( \left( 0,1,2 \right), \left( 1-R_{SCKij}-R_{CKij}, R_{SCKij}, R_{CKij} \right) \right)$$

where the status of the cow is depending on the risk of SCK (R_SCKij_), the risk of CK (R_CKij_) and the probability to be healthy (1 - R_SCKij_ - R_CKij_), respectively.

**Treatment of ketosis**

A cow with SCK has a probability of 0.1 to be treated and is treated in the same week when SCK occurred. A cow with CK is always treated immediately. The treated SCK cases (S_treat_=1) are selected randomly from a Bernoulli distribution.

**Milk production losses due to CK and SCK**

The percentage milk production losses (%MPL_ijp_) of cow *i*, in parity *p* and week *j* are different for cows with SCK and CK. Once a cow is diagnosed with SCK or CK an initial percentage is drawn from an uniform distribution, representing the percentage milk production loss of the MP_ijp_ (%MPL_SCKip_ and %MPL_CKip_)

$${\%MPL}_{ijp}=\left\{ \begin{aligned} if {SK}_{ijp}=1 and S_{treat\_ijp}=1 then \%{MPL}_{SCKip}\times{Adj}_{treat} \\ if {SK}_{ijp}=1 and S_{treat\_ijp}=0 then {\%MPL}_{SCKip} \\ if {SK}_{ijp}=2 then {\%MPL}_{CKip}\times{Adj}_{treat} \\ else {SK}_{ijp}=0 then 0 \\ i=1,\ldots,n \\ j=1,\ldots, week at drying off \\ p=1,\ldots n \end{aligned} \right.$$

When cows are treated for ketosis (S_treat_=1), there is an improvement in milk production by a treatment effect (Adj_treat_).

The production loss (MPL_ijp_) of cow *i*, in parity *p* and week *j* in the week of occurrence of either CK or SCK is highest, afterwards milk production losses decrease with an adjustment factor (AdjMPL) and is pruned to 0 when milk production losses in the previous week drop below the pruning factor Ɵ.

$${MPL}_{ijp}\boldsymbol{=}\left\{ \begin{aligned} if {\%MPL}_{ijp}>0 then {\%MPL}_{ijp}\times{MP}_{ijp} \\ if j>1 and {\%MPL}_{ijp}=0 and {MPL}_{ij-1p}>\emptyset then {MPL}_{ij-1p}\times AdjMPL \\ else {MPL}_{ijp}\leq\emptyset then 0 \\ i=1,\ldots,n \\ j=1,\ldots, week at drying off \\ p=1,\ldots, n \end{aligned} \right.$$

**Probability of conception**

The probability of conception of individual cows (PCon_ijp_) is modelled as in Inchaisri et al. (2010) and Rutten et al. (2014), assuming a base conception rate of 0.5, and adjustments based on parity (FP), relative milk production factor (FRM), and occurrence of the postpartum disorder metritis (FME). In addition, the probability of conception is also adjusted based on the occurrence of ketosis (FKET). The probability of conception was decreased by 20% when ketosis was present in the previous WIM and otherwise increased by 3% (Raboisson et al., 2014).

The FP is 1.05 and 1 for parity 1 and parity ≥2, respectively. For every cow a FRM was drawn from a normal distribution with a mean of 1.0 and standard deviation of 0.1. The FRM is 1.1, 1 and 0.9 for cows having a relative milk production <0.9, 0.9 to 1.1 and > 1.1, respectively. The FME is 0.81 in the occurrence of metritis, and 1 without the occurrence of metritis. The FKET is 0.8 when both CK and SCK occur, 0.96 when CK or SCK occur, and 1 when there is no CK or SCK.

$${PCon}_{ijp}=\left\{ \begin{aligned} \begin{aligned} if {INS}_{ijp}=0 then 0 \\ if j<5 then 0 \end{aligned} \\ else j\geq5 then \left( 41.5-345\times{0.6}^{j} \right)\times\frac{PCon\times{FP}_{ip}\times{FRM}_{ip}\times{FME}_{ip}\times{FKET}_{ip}}{0.37} \\ i=1, \ldots, n \\ j=1, \ldots,35 \\ p=1, \ldots, n \end{aligned} \right.$$

**Culling due to clinical ketosis**

The occurrence of culling due to CK is determined as a discrete event:

$${CullCK}_{ijp}=Discrete \left( \left( 1,0 \right), \left( {PCullCK}_{ijp,}1-{PCullCK}_{ijp} \right) \right)$$

where CullCK_ijp_ is the culling status of cow *i*, in parity *p* and week *j*, in which 0 indicates not culled and 1 means culled because of CK.

Cows with CK are at risk to be culled until 4 weeks after diagnosis of CK with a risk of RCullCK_ijp_ during that four week period. The probability of culling due to CK (PCullCK_ijp_) is determined as follows:

$${PCullCK}_{ijp}=\left\{ \begin{aligned} if j>1 and max {CullCK}_{ijp}:{CullCK}_{ij-1p}=1 then 0 \\ if j=1 and {SK}_{ijp}=2 then \frac{{RCullCK}_{ij}}{4} \\ if j=2 and max {SK}_{ij-1p}:{SK}_{ijp}=2 then \frac{{RCullCK}_{ij}}{4} \\ if j=3 and max{SK}_{ij-2p}:{SK}_{ijp}=2 then \frac{{RCullCK}_{ij}}{4} \\ if j\geq4 and max{SK}_{ij-3p}:{SK}_{ijp}=2 then \frac{{RCullCK}_{ij}}{4} \\ else 0 \\ i=1, \ldots, n \\ j=1, \ldots, week at drying off \\ p=1, \ldots, n \end{aligned} \right.$$

**Increased risk of displaced abomasum (DA)**. The risk of DA in the absence of ketosis (R_noDA_) and the increased risk of DA due to ketosis (R_DA_) were estimated from the annual incidence of DA and the odds ratio of DA incidence for a cow with ketosis (OR_DA_). In each time step, the probability of DA occurrence (PDA_ijp_) is determined depending on the ketosis status of the cow, and adjusted for WIM in such a way that 90% of DA cases occurred in WIM≤4 and 10% in WIM>4 (AdjWIM_DAj_). The status of DA for a cow in each time step (SDA_ij_) could be healthy (0) or having DA (1) and is simulated as a discrete event based on the probabilities of healthy (1- PDA_ij_) and having DA (PDA_ij_), respectively.

${PDA}_{ijp}=\left\{ \begin{aligned} if {SK}_{ijp}=0 and j\leq4 then R_{noDA}\times{AdjWIM}_{DA1} \\ if {SK}_{ijp}=0 and j>4 then R_{noDA}\times{AdjWIM}_{DA2} \\ if {SK}_{ijp}>0 and j\leq4 then R_{DA}\times{AdjWIM}_{DA1} \\ {if SK}_{ijp}>0 and j>4 then R_{DA}\times{AdjWIM}_{DA2} \\ i=1,\ldots, n \\ j=1, \ldots, week at drying off \\ p=1, \ldots, n \end{aligned} \right.$

$${SDA}_{ij}=Discrete \left( \left( 0,1 \right), \left( 1-{PDA}_{ij}, {PDA}_{ij} \right) \right)$$

***Increased risk of mastitis.*** Similarly as for DA, the effect of ketosis on clinical mastitis (CM) occurrence was modelled. The risk of CM in the absence of ketosis (R_noCM_) and the increased risk of CM due to ketosis (R_CM_) were estimated from the annual incidence of CM and the odds ratio of CM incidence for a cow with ketosis (OR_CM_). In each time step, the probability of CM occurrence (PCM_ijp_) is determined depending on the ketosis status of the cow, and adjusted for WIM in such a way that 70% of CM cases occurred in WIM≤4 and 30% in WIM>4 (AdjWIM_CMj_). The status of CM for a cow in each time step (SCM_ij_) could be healthy (0) or having CM (1) and is simulated as a discrete event based on the probabilities of healthy (1- PCM_ij_) and having CM (PCM_ij_), respectively.

${PCM}_{ijp}=\left\{ \begin{aligned} if {SK}_{ijp}=0 and j\leq4 then R_{noCM}\times{AdjWIM}_{CM1} \\ if {SK}_{ijp}=0 and j>4 then R_{noCM}\times{AdjWIM}_{CM2} \\ if {SK}_{ijp}>0 and j\leq4 then R_{CM}\times{AdjWIM}_{CM1} \\ {if SK}_{ijp}>0 and j>4 then R_{CM}\times{AdjWIM}_{CM2} \\ i=1,\ldots,n \\ j=1,\ldots, week at drying off \\ p=1,\ldots,n \end{aligned} \right.$

$${SCM}_{ij}=Discrete \left( \left( 0,1 \right), \left( 1-{PCM}_{ij}, {PCM}_{ij} \right) \right)$$

**Table S1:** Input values for cow factors and their relation with clinical ketosis (CK) and subclinical ketosis (SCK) for the default scenario. Also the abbreviations are mentioned for the input values. (WIM=week in milk, DA=Displaced abomasum, CM=clinical mastitis)

| **Parameters** | **Abbreviation** | **Default Value** | **Source** |
| --- | --- | --- | --- |
| Herd milk production (kg/cow/305 days) |  | 8,742 | CRV, 2017 |
| Proportion of cows in herd |  |  |  |
| Parity 1 |  | 0.32 | Inchaisri et al., 2010 |
| Parity 2 |  | 0.25 |  |
| Parity 3 |  | 0.18 |  |
| Parity 4 |  | 0.11 |  |
| Parity 5 |  | 0.07 |  |
| Parity ≥6 |  | 0.07 |  |
| Annual proportion of dairy cows with SCK | R_SCK_ | 0.11 | Van der Drift et al. 2012 |
| Annual proportion of dairy cows with CK | R_CK_ | 0.007 | Berge and Vertenten 2014 |
| Transition parameter from healthy to SCK | R_HS_ | 0.15 | Authors’ expertise |
| Transition parameter from healthy to CK | R_HC_ | 0.01 | Authors’ expertise |
| Transition parameter from SCK to CK | R_SC_ | 0.04 | Authors’ expertise |
| Annual proportion of both CK and SCK that occur WIM ≤ 4 | R_WIM≤4_ | 0.75 | McArt et al. 2012; LeBlanc 2010 |
| Annual proportion of both CK and SCK that occur WIM>4 | R_WIM>4_ | 0.25 |  |
| Treatment effect, proportion reduction in milk production losses | Adj_treat_ | 0.5 | McArt et al. 2011 |
| Adjustment factor higher risk ketosis for high producing cows  <7,600 kg per 305 days  7,601-9,000 kg per 305 days  >9,001 kg per 305 days | AdjMP | 0.85  1  1.15 | Authors’ expertise |
| Percentage milk production loss, SCK | %MPL_SCK_ | Unif1orm (6%, 8%) | Duffield et al. (2009), McArt et al. (2012), Raboisson et al. (2014) |
| Percentage milk production loss, CK | %MPL_CK_ | Uniform (15%, 17%) | Dohoo and Martin (1984) |
| Threshold level for milk production losses | Ɵ | 1.5% | Authors’ expertise |
| Adjustment factor, for the weekly decline in milk production losses | ADJ_MPL_ | 0.7 | Authors’ expertise |
| Proportion of cows culled for CK | RCull_CK_ | 0.0063 | Authors’ expertise |
| Probability of culling a cow with CK | PCull_CK_ | 0.3 | Authors’ expertise |
| Probability of culling a cow for fertility | RCull_Fert_ | 0.2 | Rutten et al. 2014 |
| Adjustment factor for general culling | ADJ_Fert_ | 1-RCull_Fert_-RCull_CK_ x R_CK_ |  |
| Annual proportion of dairy cows with DA | R_DA_ | 0.011 | Suthar et al. 2013 |
| Annual proportion of dairy cows with CM | R_CM_ | 0.3 | Lam et al. (2013); Suthar et al. (2013) |
| Odds ratio DA | OR_DA_ | 5 | Duffield et al. 2009; Berge and Vertenten (2014); Raboisson et al. 2014 |
| Odds ratio CM  Adjustment factor more DA in WIM≤ 4  Adjustment factor less DA in WIM> 4  Adjustment factor more CM in WIM≤ 4  Adjustment factor less CM in WIM> 4 | OR_CM_  AdjWIM_DA1_  AdjWIM_DA2_  AdjWIM_CM1_  AdjWIM_CM1_ | 1.3  0.225  0.002  0.075  0.015 | Berge and Vertenten 2014; Raboisson et al. 2014  Adapted from LeBlanc et al. (2005)  Adapted from Steeneveld et al. (2008) |

**References**

Berge AC, Vertenten G. A field study to determine the prevalence, dairy herd management systems, and fresh cow clinical conditions associated with ketosis in western European dairy herds. J. Dairy Sci. 2014; 97: 2145–54.

CRV. Annual report 2017. CRV (cattle breeding company), Arnhem, the Netherlands.

Dohoo I R, Martin SW. Subclinical ketosis: prevalence and associations with production and disease. Canadian Journal of Comparative Medicine-Revue Canadienne De Medecine Comparee 1984; 48:1-5.

Duffield TF, Lissemore KD, McBride BW, Leslie KE. Impact of hyperketonemia in early lactation dairy cows on health and production. J. Dairy Sci. 2009; 92: 571–80.

Inchaisri C, Jorritsma R, Vos P, van der Weijden GC, Hogeveen H. Economic consequences of reproductive performance in dairy cattle. Theriogenology 2010; 74:835-846.

Lam TJGM, van den Borne BHP, Jansen J, Huijps, K, van Veersen JCL, van Schaik G, et al. Improving bovine udder health: a national mastitis control program in the Netherlands. J. Dairy Sci. 2013; 96: 1301–11.

LeBlanc, SJ, Leslie KE, Duffield TF. Metabolic predictors of displaced abomasum in dairy cattle. J. Dairy Sci. 2005; 88: 159–70.

Leblanc S. Monitoring Metabolic Health of Dairy Cattle in the Transition Period. J. Reprod. Dev. 2010; 56:S29-S35.

McArt JAA, Nydam DV, Ospina PA, Oetzel GR. A field trial on the effect of propylene glycol on milk yield and resolution of ketosis in fresh cows diagnosed with subclinical ketosis. J. Dairy Sci. 2011; 94: 6011–20.

McArt JAA, Nydam DV, Oetzel GR. Epidemiology of subclinical ketosis in early lactation dairy cattle. J. Dairy Sci. 2012; 95: 5056–66.

Raboisson D, Mounie M, Maigne E. Diseases, reproductive performance, and changes in milk production associated with subclinical ketosis in dairy cows: A meta-analysis and review. J. Dairy Sci. 2014; 97:7547-7563.

Rutten CJ, Steeneveld W, Inchaisri C, Hogeveen H. An ex ante analysis on the use of activity meters for automated estrus detection: To invest or not to invest? J. Dairy Sci. 2014; 97:6869-6887.

Steeneveld W, Hogeveen H, Barkema HW, Van den Broek J, Huirne RBM. The influence of cow factors on the incidence of clinical mastitis in dairy cows. J. Dairy Sci. 2008; 91:1391-1402.

Suthar VS, Canelas-Raposo J, Deniz A, Heuwieser W. Prevalence of subclinical ketosis and relationships with postpartum diseases in European dairy cows. J. Dairy Sci. 2013; 96:2925-2938.

Van der Drift SGA, Jorritsma R, Schonewille JT, Knijn HM, Stegeman JA. Routine detection of hyperketonemia in dairy cows using Fourier transform infrared spectroscopy analysis of β-hydroxybutyrate and acetone in milk in combination with test-day information. J. Dairy Sci. 2012; 95: 4886–98.
